# From malaria control to elimination within a decade: lessons learned from Timor Leste, a newly independent country

**DOI:** 10.1186/s12936-020-03162-3

**Published:** 2020-03-04

**Authors:** A. M. G. Manel Yapabandara, Maria do Rosario de Fatima Mota, Raul Sarmento, Johanes don Bosco, Rajitha Wickremasinghe

**Affiliations:** 1 17/23, Hulangamuwa Road, Matale, Sri Lanka; 2 Ministry of Health, Dili, Timor-Leste; 3 Department of Public Health, Faculty of Medicine, University of Kelaniya, Ragama, Sri Lanka

**Keywords:** Malaria control, Malaria elimination, Timor Leste

## Abstract

**Background:**

Timor Leste has made remarkable progress from malaria control to malaria elimination in a span of 10 years during which organized malaria control efforts were instituted. The good practices and possible factors that have contributed to the remarkable transition from malaria control to elimination in a newly independent country devastated by civil unrest which left the entire administrative structure including the health sector in a disrupted non-functional state are highlighted.

**Methods:**

Data from the National Malaria Control Programme were reviewed. A literature search was carried out using the key words “malaria”, “Timor Leste”, “East Timor”, and “malaria control” and “malaria elimination”. All relevant manuscripts and reports that were identified in the search were reviewed. Key personnel of the NMCP, WHO and the GFATM involved in the project were interviewed.

**Results:**

With the setting up of the National Malaria Control Programme just after independence in 2003 with two officers, the programme expanded over the years and strategic malaria control activities in an organized manner commenced in 2009 with funding from the Global Fund to fight AIDS, Tuberculosis and Malaria. The incidence of malaria declined dramatically from 223,002 cases in 2006 with the last indigenous case being reported in June 2017. The decline in malaria was associated with strategic application of key evidence-based interventions taking into account the burden of disease, characteristics of vectors, and stratification of risk areas ensuring universal access to malaria prevention, and quality assured diagnostic tools and effective anti-malarial medicines at point of care, intensified surveillance, monitoring and evaluation, and capacity building, including training of staff, with adequate programme funding. The programme was provided with technical expertise and sustained political commitment that ensured uninterrupted implementation of interventions based on national strategic plans. The incorporation of the malaria control programme within an evolving health system helped the transition from malaria control to malaria elimination.

**Conclusion:**

Universal access to quality assured malaria diagnosis and treatment and focussed vector control, implemented throughout the country in an organized manner with adequate funding and political commitment were key to the successful interruption of malaria transmission in the country. All the practices or factors listed did not work in isolation but rather synergistically in an integrated manner. Malaria elimination is possible even in tropical areas of South and Southeast Asia.

## Background

Timor Leste gained independence on 20th May 2002. At the time of independence, the country had no organized administrative structure in place and did not have the capacity or the expertise to deliver healthcare services. During the early years of independence, the country was totally dependent on external resources and support to provide basic services to its population. With generous UN resources and support, the country was able to slowly develop necessary infrastructure and systems [1].

Timor Leste shares a land border with Timor Province of Indonesia which is endemic for malaria [1]. There is frequent undocumented migration through the border. In addition, nearby Indonesian islands are frequently visited by residents of Timor Leste for various purposes including fishing [2]. All of the imported malaria cases documented in Timor Leste since 2016 have been acquired in Indonesia. This complex migration pattern is a major challenge to malaria elimination in, and prevention of its re-introduction to, Timor Leste.

Even today, the health situation in Timor-Leste is far from ideal. Among the more serious problems are high infant and under-5 mortality rates caused by infectious diseases; maternal and child malnutrition is a major problem. There is a high incidence of preventable communicable diseases such as tuberculosis, childhood respiratory infections, diarrheal diseases and a rising incidence of non-communicable diseases [3]. Malaria was a major problem in the country and was ranked second in the list of top ten priority diseases 10 years ago [4]. In June 2017, the last indigenous case of malaria was reported and in 2018, 8 imported malaria cases were reported. The Malaria Elimination Oversight Committee of the World Health Organization (WHO) which met in February 2019 has indicated that Timor Leste is eligible for applying to the WHO for malaria-free certification by mid 2020.

As malaria was a major public health problem in the country at independence, the National Malaria Control Programme was established in 2003 under the newly established Communicable Disease Control Department of the Ministry of Health [4]. The National Malaria Control Programme (NMCP) was responsible for planning, implementing, monitoring and evaluation of malaria control activities in the country. At its inception in 2003, the NMCP employed two temporary national malaria control officers, who had no formal training in malaria control, and one driver funded by a GFATM round 2 Grant [4].

In the early years, the NMCP was dependent on other agencies for malaria control due to a shortage of staff, lack of infrastructure, technical expertise and funding. Even though funding from GFATM was obtained in 2003, the NMCP relied heavily on other partners and agencies that were contracted as sub-recipients to carryout malaria control activities [4]. The agencies involved included WHO, Care International, Health Net, a local NGO, Rotarians against Malaria (Australia), Global Rotary Programme, JICA and the United Nations Children’s Fund. During the early years, there was no estimate of the burden of malarial disease or a reliable data reporting system. A large number of malaria cases were detected clinically and facilities for microscopy were limited [4].

From 2006 to 2008, through a Global Fund grant, a short-term international expert malariologist was appointed to provide technical assistance to the National Malaria Control Programme; from 2009 onwards a full time international malariologist was appointed who provided technical assistance and expertise for the NMCP to design, plan and implement an evidence-based malaria control programme based on WHO guidelines. The National Strategic Plan for malaria control 2009 to 2013 was developed.

The strategies adopted and implemented during Timor Leste’s journey from malaria control to elimination and the lessons learned from this experience which may assist other countries striving to achieve malaria elimination are highlighted.

## Methods

Data from the NMCP since its inception were reviewed. The National Strategic Plans of the NMCP, GFATM grants and their reports and Annual reports sent to the WHO were reviewed. A literature search was carried out using the key words “malaria”, “Timor Leste”, “East Timor”, and “malaria control” and “malaria elimination”. All relevant manuscripts and reports that were identified in the search were reviewed. Key personnel of the NMCP, WHO and the GFATM involved in the project were interviewed.

## Results

### Burden of malaria

The NMCP commenced formal data collection in 2006. In 2006, 223,002 cases were reported in the country, the majority (185,196) being treated on clinical suspicion primarily due to limited microscopy facilities in the country. The predominant species has been *Plasmodium falciparum*. Initially, the objective was to reduce the burden of disease (4).

**Fig. 1:**
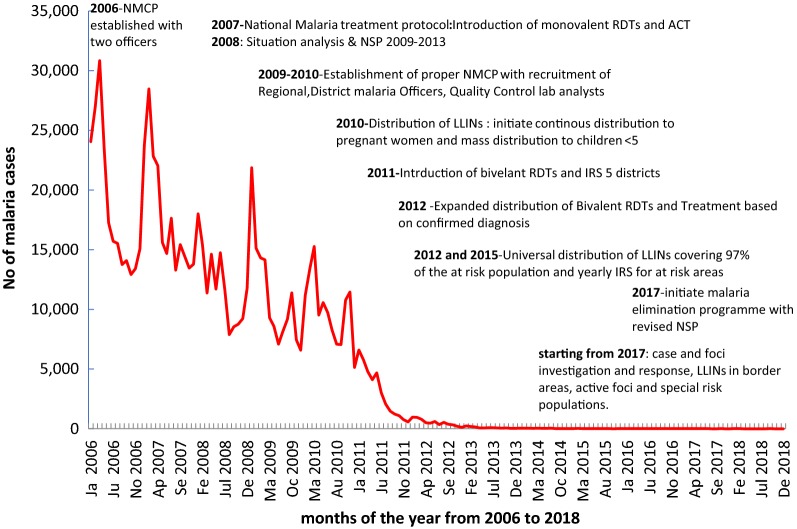
Number of malaria cases reported per month and milestones of the National Malaria Programme from 2006 to 2018

**Fig. 2:**
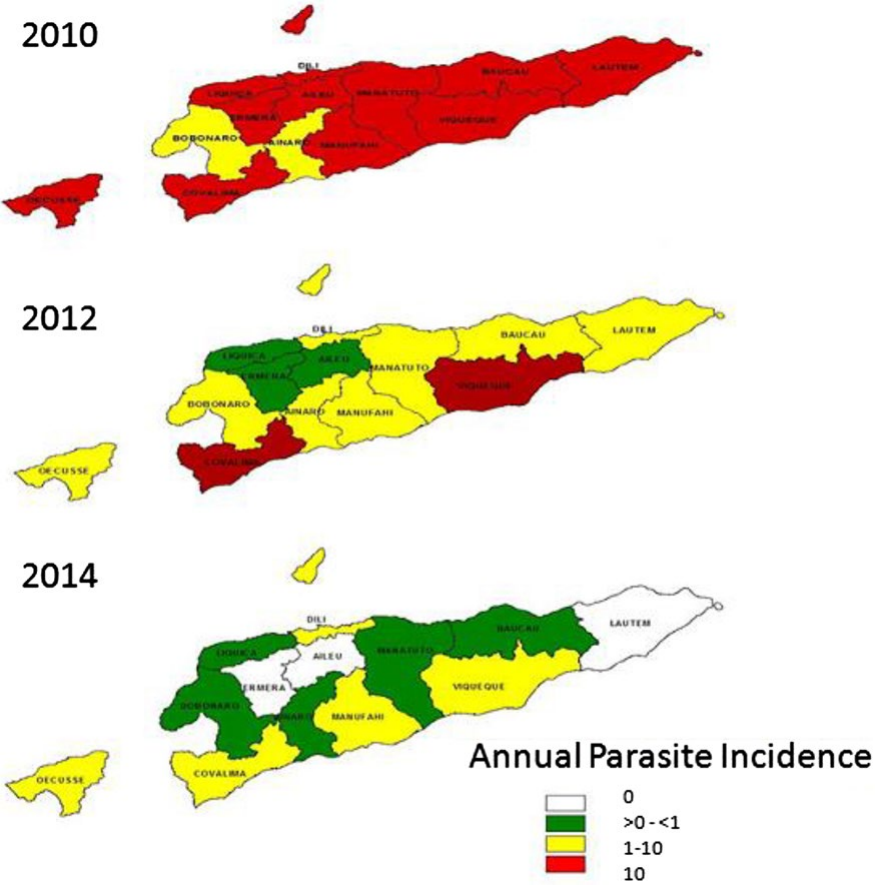
Progress from malaria control to malaria elimination district wise 2010–2014

In 2007, monovalent Rapid Diagnostic Test kits (RDT) and Artemisinin-based combination therapy (ACT) (artemether/lumefantrine) at point of care were introduced after which there was a 33% reduction in the number of cases reported and a 42% reduction in the number of clinically suspected cases in 2008 (Table 1 and Fig. 1). The next significant reduction was observed between 2010 and 2011 when there was a 70% reduction in the number cases reported and a 79% reduction in the number of cases treated on clinical suspicion. This was followed by an even greater reduction in the incidence of malaria from 2011 to 2012 with a 70% (119,072 in 2011 to 36,153 in 2012) reduction in the number of cases reported and a 94% reduction in the number of cases treated on clinical suspicion (16,418 cases in 2011 to 940 cases in 2012). Since then, there has been a gradual decline in the number of cases reported coming down to double digits in 2015 (Tables 1, 2, and Figs. 1, 2 ). In 2016, the NMCP launched the elimination programme.

**Table 1:** Number of malaria cases detected and deaths by species 2006–2018

Year	Population	Clinically treated cases	Confirmed cases	*Pf*	% *Pf*^a^	*Pv*	Mixed infections	Total	Deaths	API^b^ (/1000 pop)	Incidence (/1000 pop)
2006	1,015,187	185,106	37,896	24,219	64.44	13,477	200	223,002	58	37.33	219.7
2007	1,047,632	168,533	46,869	34,174	73.26	12,544	161	215,402	26	44.74	205.6
2008	1,080,742	97,621	45,973	34,406	75.43	11,295	272	143,594	10	42.54	132.9
2009	1,114,534	85,799	40,999	34,517	85.57	12,246	567	133,129	56	36.79	119.4
2010	1,149,028	78,822	40,250	28,350	70.82	11,432	154	119,072	58	35.03	103.6
2011	1,092,104	16,418	19,740	14,261	80.96	3759	1720	36,153	16	18.08	33.1
2012	1,118,429	940	5262	2016	56.52	2288	958	6202	4	4.70	5.50
2013	1,145,048	17	1025	373	50.05	512	140	1042	3	0.90	0.90
2014	1,172,529	5	342	118	59.36	139	85	347	1	0.29	0.30
2015	1,183,643	0	80	33	70.00	24	23	80	0	0.07	0.07
2016	1,205,067	1	94	51	89.36	10	33	95	0	0.08	0.08
2017	1,226,879	0	30	12	86.66	04	14	30	0	0.03	0.03
2018	1,249,985	0	7	4	75.00	2	2	08	0	0.006	0.006

^a^For  %*Pf* calculation, *Pf* and mixed infections were included

^b^For API calculation, only confirmed malaria cases were taken

**Table 2:** Number of malaria cases and API by district 2011–2018

District	2011		2012		2013		2014		2015		2016		2017		2018	
	Number of cases	API (per 1000 pop)	Number of cases	API (per 1000 pop)	Number of cases	API (per 1000 pop)	Number of cases	API (per 1000 pop)	Number of cases	API (per 1000 pop)	Number of cases	API (per 1000 pop)	Number of cases	API (per 1000 pop)	Number of cases	API (per 1000 pop)
Aileu	784	17.2	9	0.2	1	0.02	1	0.02	0	0.00	0	0	0	0	0	0
Ainaro	875	14.7	188	3.2	34	0.54	5	0.08	1	0.02	0	0	0	0	0	0
Baucau	4246	38.1	371	3.3	97	0.81	7	0.06	4	0.03	0	0	0	0	0	0
Bobonaro	1146	12.8	220	2.5	20	0.20	6	0.06	0	0.00	2	0.02	6	0.05	0	0
Covalima	2940	48.9	1073	17.9	75	1.17	34	0.52	5	0.07	0	0	2	0.03	1	0.01
Dili	5177	22.1	1372	5.9	265	1.05	51	0.20	5	0.02	3	0.01	10	0.03	1	0.003
Ermera	1732	15.1	96	0.8	7	0.06	4	0.03	2	0.02	1	0.01	0	0	0	0
Lautem	5484	91.1	256	4.3	30	0.47	3	0.05	0	0.00	0	0	0	0	0	0
Liquiça	290	4.6	105	1.7	18	0.26	5	0.07	0	0.00	0	0	1	0.01	2	0.03
Manatuto	1473	34.1	167	3.9	20	0.44	7	0.15	1	0.02	0	0	0	0	0	0
Manufahi	4070	83.2	278	5.7	150	2.87	41	0.77	1	0.02	0	0	1	0.02	0	0
Oecuse	2043	31.2	408	6.2	136	1.98	141	2.00	49	0.68	88	1.19	10	0.14	4	0.06
Viqueque	5893	84	1659	23.6	189	2.51	42	0.55	12	0.15	2	0.02	0	0	0	0
Total	36,153	33.9	6202	5.8	1042	0.91	347	0.30	80	0.07	95	0.08	30	0.03	8	0.006

*API* annual parasite incidence

The good practices and possible factors that have contributed to the remarkable transition from malaria control to elimination within a decade in a newly independent country devastated by civil unrest which disrupted the administrative and service structures wherein the health sector was unable to deliver healthcare services are highlighted. All the practices or factors listed did not work in isolation but rather synergistically in an integrated manner.

### Providing universal coverage with quality assured diagnostics and effective anti-malarial medicines at the point of care

There was a major reduction in the number of cases reported and the number of cases treated clinically when quality assured monovalent RDTs and ACT were introduced in 2007. These products were made available at the point of care in the public and faith-based sectors; subsequently it was extended to the private sector in 2017. In the public and the faith-based healthcare centres, the services were provided free of charge. It is likely that some of the cases previously treated on clinical grounds were in fact not malaria. The reduction in the number of cases that could be attributed to the introduction of ACT may be due to the circulating drug resistant strains of *P. falciparum* that may have been effectively treated thereby minimizing further onward transmission. Even though only monovalent RDTs were introduced initially, at least *P. falciparum* infections, the predominant malarial species, were detected that would have reduced mortality.

The reduction of the incidence of malaria was even greater with the introduction of bivalent RDTs, long-lasting insecticide-treated nets (LLINs) and selective indoor residual spraying (IRS) (Fig. 1). The bivalent RDTs distinguished *P. falciparum* and non-falciparum infections. With the expansion of diagnostic services including microscopy the number of clinically treated cases declined dramatically.

Microscopy was strengthened with the recruitment of an international laboratory technician who trained staff and strengthened the laboratory services at Community Health Centres and hospitals and ensured quality assured malaria microscopy at the National Laboratory. The microscopists are assessed regularly to maintain their diagnostic skills. Since 2014, all cases had to be confirmed by microscopy and guidelines were issued to treat only confirmed cases. An External Competency Assessment was carried out with collaboration of WHO to assess the capacity of laboratory analysts/microscopists and for selection of laboratory analysts who can be assigned for quality assurance of malaria microscopy at National and District/Municipality level.

The NMP procures only WHO pre-qualified diagnostics and anti-malarial medicines thereby providing a quality assured service. Initially, anti-malarial medicines were available in the private sector, mostly brought from neighbouring Indonesia. Currently, the Ministry of Health is the sole importer of anti-malarial medicines. There was no quality assurance of the medicines available in the private sector.

Initially, the country did not have the capacity to conduct therapeutic efficacy studies. Since 2007, *Plasmodium vivax* infections were treated with chloroquine and *P. falciparum* infections were treated with artemether/lumefantrine. Therapeutic efficacy studies done in 2015 revealed that artemether/lumefantrine was effective for treatment of *P. falciparum;* in *P. vivax* infections, there was a 17% late parasitological failure with chloroquine. Therefore, treatment for *P. vivax* was changed to artemether/lumefantrine starting from 2016.

### Having a dedicated workforce for malaria control in an evolving health system

Timor Leste was successful in securing a Global Fund round 7 grant of USD 6.47 million in 2009 for an expanded comprehensive response to sustaining malaria control in the country. This grant provided for recruitment of dedicated staff for malaria control at the national and district levels. In 2009/2010, 19 officers were recruited using GF funds at national level; at district and sub-district levels 26 and 28 personnel, respectively, were recruited. With subsequent expansion of the work force, 110 personnel were recruited between 2009 and 2011 using GFATM funding. The Programme Manager, two Regional Malaria Officers, one Vector Control Officer and 13 District/municipality malaria officers who were initially funded by GF funds were absorbed into the NMCP in 2012. After the implementation of the GFATM round10 grant programme, the NMCP had 110 staff at central/national, district and sub-district levels, cohesively functioning with the health management team within an integrated framework.

The NMP with funding from donor agencies invested on training staff. The intensive 3 months basic and re-fresher training provided to the staff with regular supervision paid rich dividends in terms of malaria control. Establishing systems in a resource poor setting was extremely difficult.

The HR and capacity development plan was revised based on the malaria control and elimination strategy. All the officers attached to the NMP were given clear TORs with job descriptions, duties in line with national malaria strategy. The availability of dedicated critical staff for malaria control in an evolving and expanding health system was a key driver of success. During this period the government invested in building capacity with foreign aid. Most of the doctors were trained in Cuba. In addition, Cuban doctors and nurses were providing in-country healthcare services; all of them were trained on the national malaria treatment guidelines annually.

**Fig. 3:**
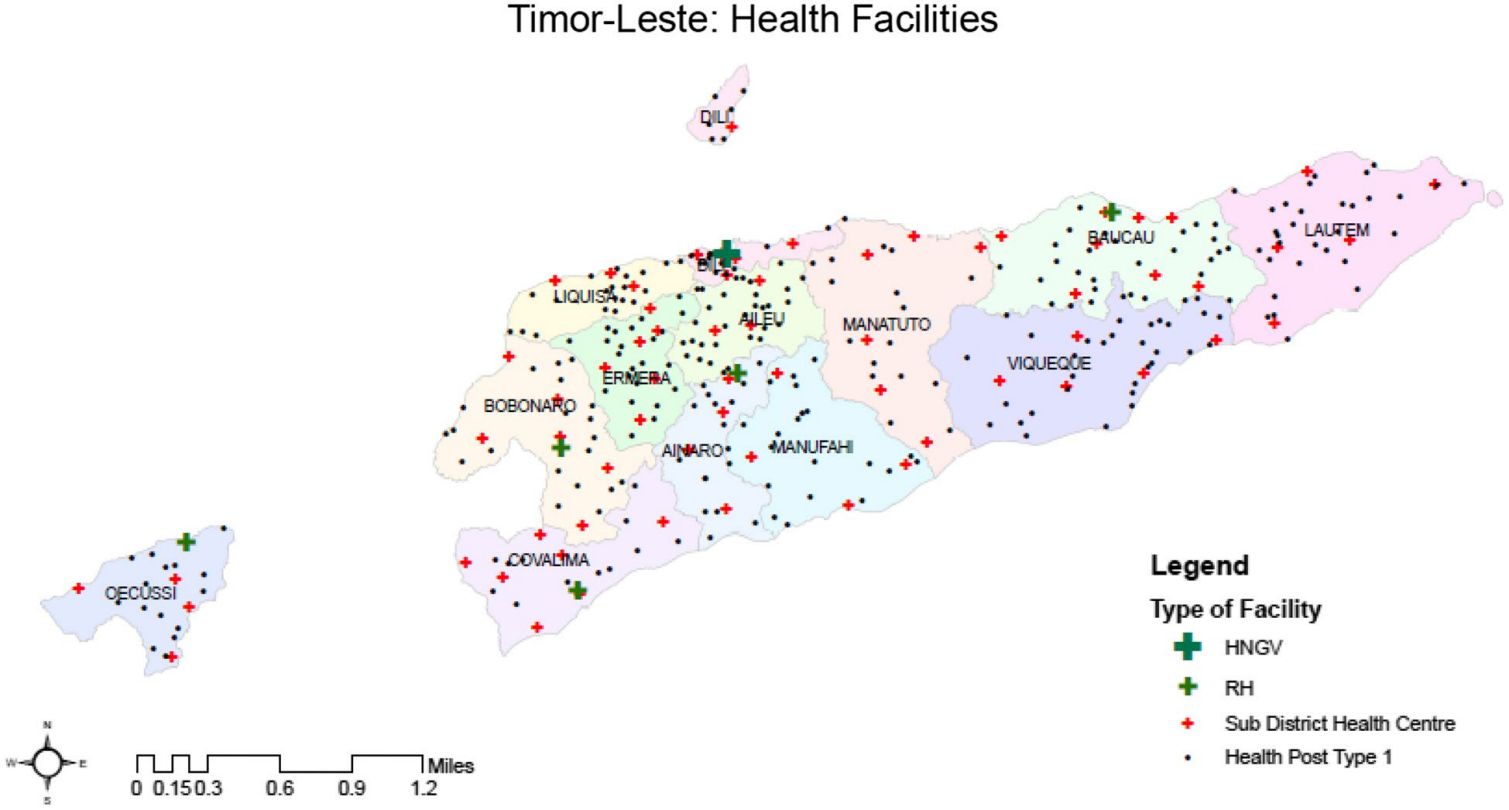
Locations of public sector health care facilities

Currently, medical care in Timor Leste is provided by 6 hospitals (one National Hospital in Dili and 5 Regional Hospitals, 69 community health centres and 273 active health posts in the public sector (Fig. 3); in addition, there are 56 active private sector providers, most of whom are located in the capital city Dili. The government plans to establish 442 health posts in the country, one for each *suco* (a group of villages). Three hundred and six health posts have been established but only 273 are currently active. Primary health care services are provided through the District Health Service structure, with Community Health Centres, Health Posts and Mobile Clinics servicing geographically defined populations within a framework of the basic services package (BSP) while incorporating SISCa (Integrated Community Health Services). Management authority and responsibility has been devolved to district health teams. Malaria diagnostic and treatment services are provided at all government healthcare institutions and during outreach services.

### Universal access to malaria prevention

A key strategy as outlined in WHO’s Global Technical Strategy 2016-2030 was providing universal access to malaria prevention as shown in Table 3 [5]. Distribution of LLINs free of charge commenced in 2006. Initially the distribution of LLINs was done by the partners and gradually the NMCP, with increase in capacity, started conducting distribution campaigns in collaboration with community leaders and Community Health Volunteers. Mass distribution of LLINs which initially focused on children under 5 was expanded to all who lived in malaria endemic districts/municipalities in 2011. Initially, one LLIN was given for two persons and later this was modified to as one LLIN per 1.8 persons. In addition, a continuous distribution programme was conducted where a LLIN was given to each pregnant female at ante-natal clinics. Assistance was provided to hang up the LLINs and their usage was monitored. The Malaria Indicator Survey carried out in 2013 reported that 97% of the population who live in malaria risk areas had slept under a LLIN the previous night. With the launching of the malaria elimination strategy in 2017, the distribution of LLINs was reduced in 2018 targeting malaria risk populations that live in the villages along the West and East Timor border areas including migratory workers, farmers engaged in slash-and-burn cultivation, armed forces and police officers, in residual non-active foci, active foci, and in Atauro island from where fisherman frequently travel to neighbouring Indonesian islands which are endemic for malaria.

**Table 3:** Malaria prevention interventions

Year	Population	LLINs distributed	Cumulative number of effective LLINs (up to 3 years)	Population protected with LLINs	Number of houses sprayed (IRS)	Population protected with IRS	% of population protected with IRS	% of population protected with LLINs
2006	1,015,187	58,153	58,153	116,306	–			11.5
2007	1,047,632	97,704	155,857	311,714	–			29.8
2008	1,080,742	79,226	235,083	470,166	–			43,5
2009	1,114,534	–	176,930	353,860	–			31.7
2010	1,149,028	170,985	250,211	450,379	12,751	70,158	6.5	39.1
2011	1,080,742	23,493	194,478	388,956	16,888	92,884	9.2	35.9
2012	1,114,534	25,143	219,621	394,670	27,288	147,031	13.2	35.4
2013	1,145,050	288,180	336,816	606,268	8082	44,451	3.9	52.9
2014	1,172,529	106,679	420,002	756,004	18,934	110,707	9.4	64.5
2015	1,183,643	27,986	422,845	761,121	20,519	102,595	8.7	64.5
2016	1,205,067	426,534	561,199	1,010,158	23,874	124,144	10.3	83.8
2017	1,226,879	197,314	623,848	1,122,926	19,195	105,576	8.6	91.5
2018	1,249,985	23,540	652,388	1,174,298	18,556	102,001	8.2	93.9

In addition to the distribution of LLINs, IRS was started in 2010 in selected districts/municipalities. Initially IRS was started in 11 sub-districts in 3 districts/municipalities namely Covalima (Maucatar, Suavi Villa, Tiliomar, Zumalai), Dili (Metinaro, Nein Feto, Christurai, Don Alexio) and Manatuto (Manatuto Villa, Nathabora and Laclo) and subsequently extended to 20 sub-districts in 10 districts based on the stratification done in 2016. IRS was carried out once a year and the insecticide used was rotated using chemically unrelated insecticides to avoid the emergence of insecticide resistance based on the WHO Global Plan for Insecticide Resistance Management in Malaria Vectors [6].

### Implementing evidence based strategies

Establishing surveillance systems, both parasitological and entomological, provided important timely information for programme management. For parasitological surveillance, a monthly reporting surveillance system was established that fed in data from each point of care. The data were compiled at sub-district/sub-municipality and district/municipality levels and transmitted to the national level. At national level, the data were checked and the final statistics were then transmitted to the HMIS.

With the reduction in the number cases, case and foci investigations and response were started in 2016 and cases were classified as imported, indigenous, introduced, induced, relapses and recrudescent based on WHO classification [7]. All cases were notified immediately by telephone and case investigation was initiated within 2 days of notification; response included entomological surveillance, mass blood surveys within 5 days and IRS being conducted within a 1.5 km radius around the residence of the reported malaria case within 10 days of notification. All data were transmitted to the national level where the information was reviewed by the national committee prior to case classification.

Regular entomological surveillance comprising adult and larval surveillance was conducted using a number of techniques by entomological teams on a monthly basis. *Anopheles barbirostris* and *Anopheles subpictus* were incriminated as primary and secondary vectors in 2010. The use of LLINs and IRS was based on the incrimination of vectors and vector bionomics. In addition, an entomological team based at national level provided oversight to the district entomological teams and was deployed in emergencies and outbreak situations.

Bioassay and bio-efficacy tests of LLINs and susceptibility tests were carried out to evaluate the efficacy and persistence of the insecticide and to evaluate the quality of IRS for corrective action for full coverage. Insecticide resistance monitoring was carried out from 2010–2018 and insecticides used for LLINs and IRS were based on the findings.

Therapeutic efficacy testing (TES) was carried out using the protocol recommended by the WHO. With the reduction in the number of cases, it was not possible to carry out TES as per the WHO protocol [8]. Integrated drug evaluation surveillance was started in 2017 after implementation of the malaria elimination strategy by integrating monitoring drug efficacy into the routine surveillance system to ensure that all patients receive the full recommended treatment under supervision and are followed up until complete cure [9].

### Quality assurance

The NMP focused on providing quality services. All malaria commodities procured were WHO prequalified products. Each batch of products was pre-tested before use. A quality assurance and quality control system was set up for malaria diagnostics including both microscopy and RDTs. All positive blood smears and RDTs were sent to the National Laboratory for confirmation by two expert microscopists and the international QA technician attached to the National Laboratory, and feedback was given to the relevant analysts. In addition, 10% of negative blood smears was also sent for confirmation. Laboratory analysts who perform microscopy and RDT examination were assessed regularly with regard to the quality of the blood smear, staining of the blood smear and accuracy of diagnosis. Laboratory analysts (microscopists) who under-performed were re-trained. In addition, regular in-service training programmes were conducted for laboratory analysts. An external competency assessment of malaria microscopy has been conducted once every 2 years since 2009.

Before each cycle of IRS, spray machine operators were trained on proper methods of application and spray machines were calibrated. All spray activities were closely monitored and bioassay tests carried out regularly as previously mentioned.

All malaria control activities were supervised regularly by District/Municipality, sub-district/sub-municipality and Regional Malaria Officers, and Regional Vector Control Officers at the national and municipality/district levels for quality of spraying and to ensure > 80% coverage of households sprayed with insecticides. In addition, a monitoring and evaluation framework, as required by the Global Fund, was established and strictly adhered to.

### Having an effective behavioural change communication (BCC) strategy

BCC and community mobilization are an integral part of the national malaria strategy providing cross cutting support for the implementation of different strategies to prevent, control and eliminate malaria. A BCC strategy was developed for the period 2015–2020. The Malaria Indicator and Health Facility survey carried out in 2013 revealed that 89.2% of pregnant women and 88.8% children under 5 years had slept under a LLIN the previous night. Most women were aware that LLINs are an effective malaria prevention method. 75% of the children had taken treatment from a health facility or health worker within 48 h of the onset of symptoms and over 90% within 3 days of onset of fever. Most women had knowledge of malaria symptoms and prevention methods. The government health facilities have been the major provider of healthcare and health related information.

### Planning of activities

The NMCP developed costed national strategic plans based on situational and gap analyses since 2009 [10], and recommendations given by the external reviews carried out in 2010, 2013 and 2017, giving a clear vision of the financial resources required. Key performance frameworks were developed and indicators identified at national, district/municipality and sub-district/sub-municipality levels. These frameworks were subject to regular audits which ensured regular monitoring of activities. As GF funding was performance-based there was motivation for ensuring activities were carried out as planned.

Quarterly meetings were held with the National and District Malaria Officers and other national officers and semi-annual meetings at district/municipality levels were conducted to monitor progress.

### Availability of funding

The NMCP since its inception has been largely funded by GFATM (Table 4). Other substantial donors include WHO, Rotarians Against Malaria (Australia), the Global Rotary Programme, Care International, JICA and UNICEF. The availability of funds ensured uninterrupted implementation of all planned activities. This was important to maximize the gains achieved over many years of application of sustained evidence-based malaria control activities.

**Table 4:** Contribution of Global Fund grants to the National Malaria Programme of Timor Leste

Grant number	Amount	Duration
TMP-202-G01-M-00	$ 2,734,774	01 Sep 2003–31Aug 2006
TMP-709-G05-M	$ 4,698,114	2009
	$ 2,367,459	2010
	$3,902,652	2011
	$ 5,375,143	2012
	$2,604,409	2013
	$2,981,432	2014
TLS-M-MOH	$2,610,355	2015
	$3,264,859	2016
	$4,039,622	2017
	$ 1,640,396	2018

### Organization of the National Malaria Programme

The organizational structure the National Malaria Programme (the name was changed from the national malaria control programme once the programme moved into the elimination phase) was essentially a vertical programme within the Department of Communicable Diseases of the Ministry of Health integrated with clinical and diagnostic services provided by a network of institutions spread throughout the country. Integration with the clinical and diagnostic services ensured that people had access to quality assured malaria diagnostics and treatment at the point of care. The vertical programme also ensured trained dedicated key staff was in place to concentrate only on anti-malarial activities. It also created a sense of accountability for the programme as otherwise competing priorities may have compromised the dedication solely to malaria control.

### Political will and cooperation

The vision of the Ministry of Health of Timor Leste is “Healthy East Timorese people in a healthy East Timor” embracing the notion that health problems are not the sole concern within the boundaries and responsibility of the health sector [11]. Over the years, the Ministry of Health has been adopting a series of policies to provide improved healthcare services to the community at the *suco* level through sustained political commitment at the highest level. The NMCP used this opportunity to provide universal access to malaria diagnosis and treatment at all points of care which enabled early diagnosis and treatment of cases minimizing the transmission potential of infected persons.

### Provision of technical assistance

The technical inputs given by the WHO through funding from GF by providing dedicated expert staff for malaria control/elimination in the country was a major impetus for the malaria control programme. It ensured that globally accepted principles of malaria control, elimination and prevention of re-introduction of malaria were adopted and practised. It also ensured that a young team of professionals in a developing country are trained adequately in the control, elimination and prevention of re-introduction of malaria.

## Discussion

Timor Leste’s transition from malaria control to elimination in a newly independent country is remarkable given the meagre resources and infrastructure the NMCP had at its inception. There are many lessons to be learned from this achievement which could guide other countries aspiring to eliminate malaria.

A major reason for the dramatic reduction in the incidence with the last indigenous case of malaria being reported in June 2017 was ensuring universal coverage of quality assured malaria diagnosis and treatment services at the point of care and of malaria prevention. The vertical NMCP integrated with clinical and diagnostic services provided through a network of institutions spread throughout the country enabled provision of universal coverage of diagnosis and treatment services at the point of care. Having dedicated staff for malaria control ensured provision of universal coverage for malaria prevention through LLINs and IRS complemented with an effective BCC programme; malaria indicator surveys revealed high uptake and usage of prevention methods. The introduction of IRS in addition to use of LLINs was based on the epidemiological profile of malaria, vulnerability and receptivity based on entomological surveillance.

Having dedicated essential staff for malaria control in an integrated system is key to a successful malaria control programme. The success story of Sri Lanka provides ample evidence for this. Sri Lanka maintained the Anti Malaria Campaign at the central level but provided malaria elimination and prevention of re-introduction services through a devolved Provincial Health Directorate [12, 13]. The Anti Malaria Campaign in Sri Lanka provided technical guidance and coordinated malaria control activities at national level [12, 13]. The added benefit of this system was the ability of the Ministry of Health, Nutrition and Indigenous Medicine of Sri Lanka to focus on malaria elimination and prevention of re-introduction in a scenario where dwindling cases made the disease “invisible” with the possibility of diverting resources to other public health priorities as a consequence [12, 13].

Malaria elimination and prevention of its re-introduction/re-establishment is not cheap. The availability of funding shows that malaria elimination can be achieved even in a low-income tropical country with a poorly developed but evolving functional health system. The availability of financial resources was able to ensure quality assured anti-malarial commodities, trained human resources, universal access to malaria diagnosis, treatment and prevention, technical support, close monitoring and evaluation, and overall management of services, all of which are highlighted in the Global Technical Strategy for Malaria 2016–2030 [5].

Although Timor Leste was fortunate to receive generous financial assistance from GFATM and other donor agencies in the past, the major challenge to prevention of re-introduction/re-establishment of malaria in the future will be the ability of the Government to provide quality assured anti-malarial services with a myriad of competing priorities in the transition phase of GFATM funding and beyond. Although elimination of malaria from Timor Leste is laudable, the efforts will have to be sustained until at least elimination of malaria in neighbouring Indonesia, and even further, till the elimination of malaria in the region and global eradication of malaria.

The establishment of the NMCP just after gaining independence had a major contribution to malaria being on the radar of the Ministry of Health. Its presence ensured that malaria was considered an important priority at all stages of the evolving health system of the country.

The elimination of malaria in the country also raises two important questions 1) what priority prevention of re-introduction/re-establishment of malaria should be given in the future; 2) when should the general public health services of the country take over prevention of re-introduction/re-establishment of malaria activities. The answer to the first question is that priority for prevention of re-introduction/re-establishment of malaria should be very high at least until neighbouring Indonesia, in particular Timor province, eliminates malaria. The answer to the second question is more difficult: despite Sri Lanka even after achieving malaria-free certification in 2016 and maintaining a dedicated work force for anti-malarial work within a devolved health system reported an introduced malaria case in December 2018; however, the existence of the dedicated work force for anti-malarial activities ensured that no further spread of malaria occurred through intensive coordinated efforts [14]. It will be wise for the Ministry of Health of the Democratic Republic of Timor Leste to maintain the existing programme structure till at least malaria-free certification and perhaps even a few years beyond; the re-orientation of the programme should be carefully considered and well planned as it shares a land border with Indonesia where malaria is endemic, and be prepared for any eventuality if malaria tries to be re-establish itself. Later the NMCP may be integrated as a vector borne disease control programme by assisting the dengue control programme in the country as dengue is a major public health problem in the country.

While political will and cooperation have been expressed and provided for the implementation of the national strategic plan for malaria, the commitment of providing financial and human resources for the NMCP by the government has been poor. As the country transits from a malaria elimination phase to a prevention of re-introduction/re-establishment phase, two transitions have to be made: (1) a technical transition from a malaria elimination mode to a prevention of re-introduction/re-establishment mode; and (2) a financial transition from donor funding to government funding. The change in the technical strategy will be minor as the country has a land border, through which undocumented migration occurs, with a malaria endemic area which will require the same amount vigilance to be maintained as currently practised. As the current programme is largely funded by a transition grant from the GFATM (approximately 60% of direct costs excluding the clinical services and salaries for laboratory analysts), the major transition will have to be the financial one where the government will have to fund the entire NMCP in the future. Failure to do so may result in a resurgence of malaria as was experienced by Sri Lanka in the 1960s (5).

## Conclusions

Malaria elimination from Timor Leste is a major public health achievement in the region and globally. Its significance viewed from a low-income tropical country lens is even greater. Good evidence-based public health practice with secure financial resources was the cornerstone of this achievement. A combination of effective interventions provided by a dedicated team ensured that the task was achieved.

This landmark achievement is a testimony that malaria can be eliminated even in developing tropical countries and should be an impetus for countries aspiring to eliminate malaria. The challenge Timor Leste now faces is the prevention of re-introduction and re-establishment of malaria; it will depend on ensuring adequate funding to implement a sustainable and effective programme, the failure of which will result in the gains achieved thus far disappearing very quickly.

## Data Availability

The data generated and/or analysed in this publication are not publicly available due to the fact that they belong to the Ministry of Health, Timor Leste. Clarifications regarding data can be made through Maria do Rosario de Fatima Mota the current programme manager and Raul Sarmento, former programme manager who are authors of this publication.

## Abbreviations

ACT: Artemisinin-based combination therapy
API: Annual parasite incidence
BCC: Behavioural change communication
BSP: Basic services package
CHC: Community Health Centre
GF: Global Fund
GFATM: Global Fund to fight AIDS, Tuberculosis and Malaria
HMIS: Health Management Information System
IRS: Indoor residual spraying
JICA: Japan International Coorperation Agency
LLIN: Long-lasting insecticidal nets
NMCP: National Malaria Control Programme
NMP: National Malaria Programme
QA: Quality assurance
RDT: Rapid diagnostic test
SISCa: Integrated Community Health Services
TES: Therapeutic efficacy study
UN: United Nations
UNICEF: United Nations Children’s Fund
USD: United States dollar
WHO: World Health Organization
